# Specific exercises performed in the period of brace weaning can avoid loss of correction in Adolescent Idiopathic Scoliosis (AIS) patients: Winner of SOSORT's 2008 Award for Best Clinical Paper

**DOI:** 10.1186/1748-7161-4-8

**Published:** 2009-04-07

**Authors:** Fabio Zaina, Stefano Negrini, Salvatore Atanasio, Claudia Fusco, Michele Romano, Alessandra Negrini

**Affiliations:** 1 ISICO Milano(Italian Scientific Spine Institute), Via Bellarmino 13/1, 20141 Milan, Italy; 2 ISICO Vigevano, Corso Pavia 37, 27029 Vigevano (PV), Italy

## Abstract

**Background:**

Exercises are frequently performed in order to improve the efficacy of bracing and avoid its collateral effects. Very frequently there is a loss of correction during brace weaning in AIS treatment.

**Aim:**

To verify the efficacy of exercises in reducing correction loss during brace weaning.

**Study Design:**

Retrospective controlled study.

**Population:**

Sixty-eight consecutive patients (eight males), age 15 ± 1 and Cobb angle 22 ± 8° at start of brace weaning.

**Methods:**

The start of brace weaning was defined as the first visit in which the wearing of brace for less than 18/24 hours was prescribed (according to our protocol, at Risser 3). Patients were divided into two groups according to whether or not exercises were performed: (1) EX (exercises), included 39 patients and was further divided into two sub-groups: SEAS (who performed exercises according to our institute's protocol, 14 patients) and OTH (other exercises, 25 patients) and (2) CON (controls, 29 patients) that was divided into two other sub-groups: DIS (discontinuous exercises, 19 patients) and NO (no exercises, 10 patients). Complete brace weaning was defined as the first visit in which the brace was no longer prescribed (ringapophysis closure or Risser 5, according to our protocol).

ANOVA and Chi Square tests were performed.

**Results:**

There was no difference between groups at baseline. However, at the end of treatment, 2.7 years after the start of the weaning process, Cobb angle increased significantly in both the DIS and NO groups (3.9° and 3.1° Cobb, respectively). The SEAS and OTH groups did not change. Comparing single groups, OTH (with respect to DIS) had a significant difference (P < 0.05).

**Conclusion:**

Exercises can help reduce the correction loss in brace weaning for AIS.

## Background

Specific exercises alone or during bracing [1-5] are used in many countries in adolescent idiopathic scoliosis (AIS) treatment; nevertheless, some authors question this approach.[6] Specific exercises are recommended by the Italian guidelines for conservative treatment of idiopathic scoliosis[7] and by SOSORT guidelines[8] as the first step in the treatment of AIS, and known systematic reviews have confirmed their effectiveness.[9] A recent study by our group showed a significant reduction in brace prescriptions thanks to specific exercises in AIS patients at risk for brace treatment.[10]

Exercises in association with bracing are considered differently by the existing schools. For some physicians they must be done independently of the brace (e.g. the same exercises are performed for patients whether or not the brace is worn),[3,11] while for others specific exercises should be designed to help the brace action. [12-17] According to the latter, during brace treatment specific exercises are recommended as the means to avoid collateral effects of bracing such as spinal stiffness and muscular strength loss, as well as to improve brace efficacy.[7] Exercises are specifically designed and proposed in order to reach definite goals during different phases of treatment: For example, mobilizing exercises performed continuously for two months in preparation to brace wearing showed better results at five months than non-specific exercises,[18] while exercises performed with the brace-on allowed adjunctive forces to be applied.[15] Until now no results have been ever published on the effectiveness of exercises in avoiding correction loss during the weaning period.

The effectiveness of exercises during brace weaning can be (at least partially) the reason for the poor results reported by Dolan in her recent metanalysis of the English literature on bracing alone (without exercises).[19] In fact, using the same inclusion criteria,[19] the papers published by some members of the international Scientific Society on Scoliosis Orthopaedic and Rehabilitation Treatment (SOSORT), [20-24] but also presented in the English literature with the inclusion of exercises, gave totally different results.[25] It is then possible that exercises play a major role in brace treatment, or that they play a role in increasing correction[18] or limiting the loss of correction.

Brace weaning is a critical phase of AIS treatment that has not been standardized. Some experts use to reduce the daily hours of brace in a somewhat rapid way, shifting from full-time wearing (20 or more hours per day) to the point that the patient was totally free from the brace within a period of six to 12 months. Others progressively and slowly reduce the hours of brace use, with a mean reduction of two to three hours every six months.[2] During this phase the trunk's ability to bear itself without any external device (the brace) could be relevant.[26] Therefore, exercises could play a role in training the spinal muscles and reduce the sort of postural collapse[27,28] that can erase part of the results achieved during brace treatment. A loss of correction during brace weaning in AIS treatment is, in fact, quite frequent and could be related to the aforementioned factors. Nevertheless, at the moment no data is available regarding a better way to wean a patient from the brace, or about the effects of exercises during this step.

The aim of the present study was to verify the efficacy of exercises in reducing correction loss during brace weaning.

## Methods

### Study design

This is a retrospective controlled study on the prospective ISICO database, including all patients treated by the institute since 2003. The data was collected during everyday clinical practice.

### Population

Sixty-eight patients (eight males) falling within the inclusion criteria, age 15 ± 1 years and Cobb angle 22 ± 8° at start of brace weaning (Table 1). The inclusion criteria were as follows: AIS under brace treatment, Risser sign 3 or more according to the French version of the sign.[29] Patients were divided into two groups according to whether or not exercises were performed: (1) EX (exercises), included 39 patients and was further divided into two sub-groups: SEAS (who performed exercises according to our institute's protocol, 14 patients) and OTH (other exercises, 25 patients) and (2) CON (controls, 29 patients) that was divided into two other sub-groups: DIS (discontinuous exercises, 19 patients) and NO (no exercises, 10 patients).

**Table 1:** Population characteristics at the start of brace weaning (T0).

Group	N° patients (females)	Age (years)	Cobb Angle	ATR	Risser
SEAS	14 (12)	14 ± 1	21.3° ± 10.4	6.0° ± 3.5	3

OTH	25 (24)	15 ± 2	22.9° ± 6.8	4.5° ± 2.7	3

DIS	19 (16)	16 ± 1	22.1° ± 9.7	4.3° ± 2.6	3

NO	10 (8)	14 ± 1	19.2° ± 5.2	7.0° ± 6.3	3

SEAS: patients who performed exercises according to our Institute protocol; OTH: patients who performed other exercises; DIS: patients who performed exercises discontinuously; NO: patients who never performed exercises. No statistically significant difference were present at T0.

#### Exercise Group (EX)

##### SEAS sub-group

Patients performing exercises according to our institute protocol.[30] SEAS is the acronym for "Scientific Exercise Approach to Scoliosis." It was coined about 30 years ago, but it was continuously updated due to scientific advancements that were achieved over the years. Individually adapted exercises are taught to the patients in our institute during a single session of 1.5 hours every two to three months: An expert physiotherapic evaluation is performed in order to develop a customized exercise protocol, a TV record with physiotherapist's suggestions is given to the patient together with the exercise protocol, and family counselling is given in regard to scoliosis treatment and management. The patient continue treatment by himself or herself at home or at a rehabilitation facility near home twice a week (40 minutes) plus one daily exercise (five minutes). The SEAS exercises during brace weaning are progressively similar to those performed in order to avoid the brace according to the hours per day allowed without the brace.[2] At 18 hours of brace wearing, half the exercises are still performed with the brace on, while at 12 hours of bracing nearly all of them are performed without the brace. With the brace on, exercises are done in order to increase the pushing of the brace and recreate the physiological kyphosis.[15] Contrastingly, those without the brace are based on Active Self-Correction (ASC), which is an active movement performed to achieve the maximum possible correction and thereby activate neuron-motor reflex corrective answers.[10,31] In all phases, therapists avoid increasing the spine's range of motion (while in any case maintaining it), as instead they focus mainly on spinal stabilization.[31]

##### OTH sub-group

The participants performed many different exercise protocols at a local facility according to what was preferred by their particular therapist.[10] In most cases these were in a group context, while in all cases the exercises lasted 45 to 90 minutes and were performed two or three times per week. Some of the patients were required to repeat the exercises daily at home.

#### Control Group (CON)

##### DIS sub-group

It was made up of patients who did not perform exercises regularly (less than 45 minutes per week, or more than six months without exercising).

##### NO sub-group

It was made up of patients who did not perform exercises.

The exercise time was calculated on the basis of what was declared at each particular visit by the patients and family.

### Protocol

Start of brace weaning was defined as the first visit during which it was prescribed that a brace be worn for less than 18/24 hours. At this stage an initial x-ray without the brace within six hours was made and, according to our bracing protocol, weaning started if Risser 3 stage was reached despite the patient's age. All the patients gradually reduced the daily hours of brace usage by two to three hours (according to clinical and/or radiographic evaluations) over six-month intervals until the prescription reached eight hours nightly, and then stopped after six months. An x-ray evaluation without the brace after 48 hours (final x-ray) was made. The mean duration of the brace-weaning phase was two years and seven months, with no differences evident among the groups and sub-groups. Complete brace weaning was defined as the first visit in which the brace was no longer prescribed.

### Data analysis

Cobb angles and ATR (angle of trunk rotation, Bunnell degrees) of the major curvature were evaluated at the start and after complete brace weaning, and we considered the mean values of these parameters. We also analysed the number of patients clinically changed at a significant level, setting the cut off at 5° for Cobb angles and 3° Bunnell for ATR (minimum significant change for each measurement and according to SRS criteria)[32,33] The paired ANOVA (with post-hoc analysis) was made to compare Cobb degrees and ATR, while Chi Square testing was performed in order to compare the results among the groups.

This study was conducted in compliance with the SOSORT criteria.[25]

## Results

**Figure 1:**
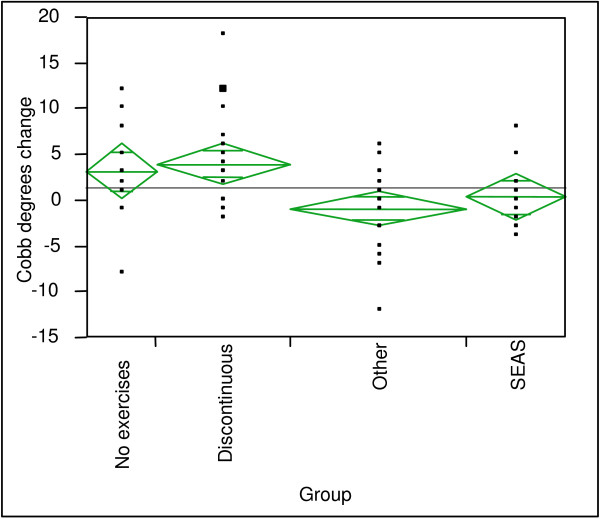
**Group comparison after brace weaning**. The figure shows the changes in Cobb angle at the end of treatment.

There were no differences between groups at baseline (T-1) nor at the first brace prescription (T0; Table 2). At the end of treatment, CON worsened by 3.25° Cobb with respect to EX (0.20°). This differential was statistically significant, but we found no difference for ATR. the DIS and NO sub-groups worsened significantly (3.9° and 3.1° Cobb; p < 0.05), while SEAS and OTH did not change (Fig. 1). Comparing individual sub-groups, only OTH was significantly different from DIS (P < 0.05). Again, changes of ATR were not significant among or within the sub-groups.

**Table 2:** Population characteristics at first brace prescription (T-1), at the start of brace weaning (T0) and at the end of treatment (T1).

	Cobb Angle			ATR (Bunnell degrees)		
Group	T-1	T0	T1	T-1	T0	T1

SEAS	25.4° ± 8.9	21.3° ± 10.4	21.6° ± 9.4	9.2° ± 10.6	6.0° ± 3.5	6.9° ± 4.3

OTH	26.8° ± 6.5	22.9° ± 6.8	22.0° ± b6.9	7.8° ± 3.7	4.5° ± 2.7	4.6° ± 3.9

DIS	29.4° ± 8.9	22.1° ± 9.7	26.1° ± 9.7	9.9° ± 5.1	4.3° ± 2.6	5.4° ± 4.3

NO	23.6° ± 8.9	19.2° ± 5.2	22.3° ± 7.3	9.6° ± 4.6	7.0° ± 6.3	7.4° ± 7.1

SEAS: patients who performed exercises according to our institute's protocol; OTH: patients who performed other exercises; DIS: patients who performed exercises discontinuously; NO: patients who never performed exercises. There were no statistically significant differences among groups at T-1, T0, T1. For Cobb angles and ATR there were statistically significant differences from T-1 to T0, from T0 to T1 and from T-1 to T1 except from T1 to T0 for ATR.

**Figure 2:**
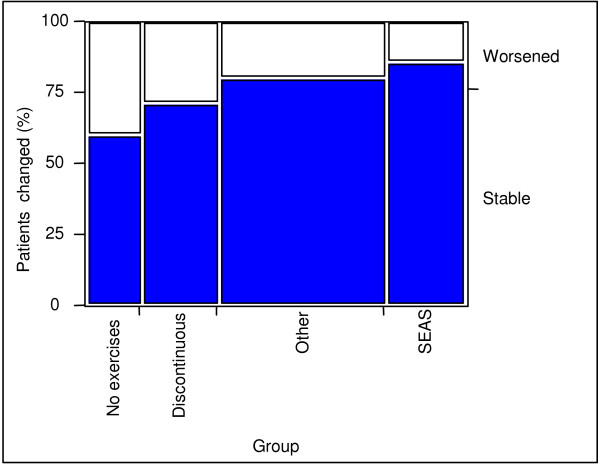
**Patients with clinically significant change in Cobb angle**. Patient change: percentage of patients with more than 5° Cobb angle change.

**Figure 3:**
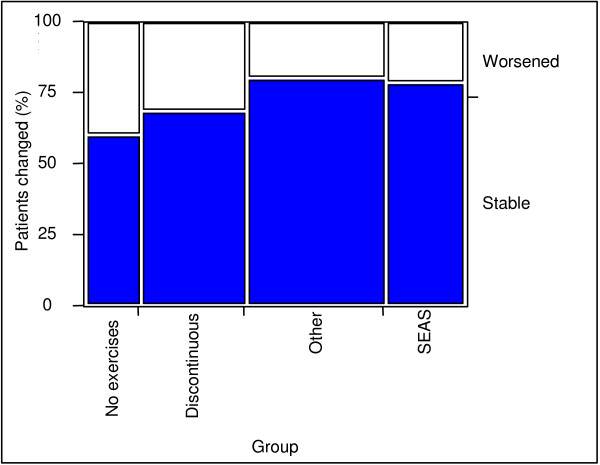
**Patients with clinically significant ATR change**. Patient change: percentage of patients with more than 3° Bunnell change.

In terms of Cobb angles, 14.3% of the patients in the SEAS group worsened (percentage of patients who had an increase of more than 5° Cobb, according to SRS criteria[33]) with respect to 20% of OTH, 28% of DIS and 40% of NO (Fig. 2). However, the Chi-square test failed because some groups were too small. In terms of ATR there was no difference among the EX groups, while the CON groups had a more relevant worsening (SEAS 21%, OTH 20%, DIS 32%, NO 40%. We considered a change of more than 3° Bunnell (Fig. 3) to be significant. The latter two parameters did not reach a significant level in Chi-square testing because some groups were too small.

## Discussion

Specific exercises are effective in reducing the loss of correction during brace weaning. Patients performing exercises according to the SEAS protocol were able to achieve the best results in clinical terms, with the lowest percentage of patients worsening (only one patient exceeded 5° Cobb). Those patients who never performed exercises (NO sub-group) had the worst results, but continuity also proved to be important (DIS sub-group). In five cases, (all in the NO and DIS sub-groups) an impressive loss of correction was seen, over 10° Cobb, and in one case even of 18° Cobb was achieved. The effect on the prominence (ATR) was less evident, and it appears this is driven more by the brace than whether exercises were performed or not.

According to these results, we can assume that exercises can prevent a sort of postural collapse[27,28] becoming evident when the spine is progressively released from the passive support of the brace. Wearing a brace for many years can cause progressive muscular atrophy and loss of strength.[12,26] If the spinal muscles are not constantly and specifically trained throughout the treatment and particularly during brace weaning, this postural collapse could be more dramatic. Our results support this hypothesis, even if more studies are needed to understand whether an increase of compliance (that was not possible to test accurately in this study) can be implied[25]

The benefit of exercises on ATR stabilization is less evident. ATR is a measure of the deformity. We can aesthetically model the trunk in general and the prominence with braces by pushing on prominences, and the results on aesthetics at the end of brace treatment are significant and persistent[34,35] Apparently, exercises are not able to influence the possible (in any case reduced – on average 0.5° ATR) loss of correction of ATR. It seems that exercises are not as effective on this bone remodelling as they are on other parameters that could be more related to posture (i.e. loss of correction).

Exercises have already been shown to be effective in the preparation for bracing, during the correction phase and while the brace is worn[15,18,36] All the exercises studied in association with bracing in our previous papers have been developed to help the brace action function properly (in a way confirming the importance of specific developed exercises, as proposed by our and other schools,[2,12] while in this case exercises during the brace-weaning phase are similar to those performed without the brace, in order to avoid postural collapse.[10]

This is the first study that has documented the efficacy of exercises in reducing loss of correction during a truly relevant phase of treatment, namely brace weaning. We are not aware of any previous study on this topic, so we do not have other data against which our results could be compared.

## Competing interests

The authors declare that they have no competing interests.

## Authors' contributions

All authors made substantial contributions to conception, design and acquisition of data; they have been involved in drafting and revising the manuscript; they have given final approval of the version to be published.
